# Supplementary material for: The Impact of the COVID-19 Pandemic on Pediatric Microbial Resistance Patterns and Abandonment Rates in Western Romania—An Interdisciplinary Study
Source: Antibiotics (Basel). 2025 Apr 16;14(4):411. doi: 10.3390/antibiotics14040411 (PMC12024448; doi:10.3390/antibiotics14040411)
Supplement: Supplementary file 1 [file antibiotics-14-00411-s001.zip › supplement S1- demographics and pathogen identification.pdf]

Category	Class	Agent
Antibiotics	Penicillins	Amoxicillin
		Ampicillin
		Oxacillin
		Penicillin
		Piperacillin
		Ticarcillin
		Methicillin
	Cephalosporins	Cefepime
		Cefotaxime
		Cefoxitin
		Ceftazidime
		Ceftibuten
		Ceftizoxime
		Ceftriaxone
		Cefuroxime
	Combination therapy- Penicillins and Cephalosporins	Amoxicillin/ Clavulanic Acid
		Ampicillin / Sulbactam
		Piperacillin/ Tazobactam
		Ticarcillin/ Clavulanic Acid
		Ceftazidime/ Avibactam
	Carbapenems	Ertapenem
		Imipenem
		Meropenem
	Fluroquinolones	Ciprofloxacin
		Levofloxacin
		Moxifloxacin
		Norfloxacin
		Ofloxacin
	Aminoglycosides	Amikacin
		Gentamicin
		Gentamicin- High Level
		Kanamycin
		Neomycin
		Netilmicin
		Streptomycin- High Level
		Tobramycin
	Macrolides	Azithromycin
		Clarithromycin
		Erythromycin
	Cyclines	Tetracycline
		Minocycline
		Tigecycline
	Glycopeptides	Teicoplanin
		Vancomycin
	Urinary	Nalidixic Acid
		Fosfomycin
		Nitrofurantoin
	Lincosamides	Clindamycin

Table S1.1. List of antimicrobials used for AST

	Reserve/ last resort	Clindamycin- Inducible<br>Linezolid<br>Colistin
	Other	Trimethoprim/ Sulfamethoxazole<br>Fusidic Acid<br>Chloramphenicol<br>Rifampicin<br>Aztreonam
Antifungals	Polyenes	Amphotericin B<br>Nystatin
	Azoles	Clotrimazole<br>Econazole<br>Fluconazole<br>Itraconazole<br>Ketoconazole<br>Miconazole<br>Voriconazole
	Other	Flucytosine<br>Caspofungin

Table S1.2. Patient demographic data

		**2019**	**2021**	**2023**	**Total**	**Chi 2**
Patients (n,%)		1417 (40.14%)	765 (21.67%)	1489 (42.18%)	3530	NA
Samples (n,%)		2483 (36.06%)	1672 (24.28%)	2730 (39.65%)	6885	NA
Sex	Male	725 (51.16%)	412 (53.86%)	747 (50.17%)	1809 (51.25%)	0.2489
	Female	693 (48.84%)	353 (46.14%)	742 (49.83%)	1721 (48.75%)	
Location of residence	Urban	809 (57.09%)	417 (54.51%)	756 (50.77%)	1896 (53.71%)	0.0028 *
	Rural	608 (42.91%)	349 (45.49%)	733 (49.23%)	1634 (46.29%)	
Age group (Chi²)	Infant	507 (35.78%)	307 (40.13%)	518 (34.79%)	1332 (37.73%)	0.0334 *
	Preschool	359 (25.34%)	172 (22.48%)	377 (25.32%)	908 (25.72%)	
	School	357 (25.19%)	168 (21.96%)	401 (26.93%)	926 (26.23%)	
	Adolescent	200 (14.11%)	123 (16.08%)	199 (13.36%)	522 (14.79%)	
Age (Kruskal-Wallis)	Median	3	3	3	3	0.1271
	IQR (value)	8	10	8	9	
	IQR (interval)	0-8	0-10	0-8	0-9	
**IRR (95% CI; p)**		**2019 vs. 2021**	**2019 vs. 2023**	**2023 vs. 2021**		
Patients		1.85 (1.70-2.03; <0.0001) *	0.95 (0.88-1.02; 0.1816)	1.95 (1.78-2.13; <0.0001) *	NA	
Samples		1.49 (1.40-1.58; <0.0001) *	0.91(0.86-0.96; 0.0006) *	1.63 (1.54-1.74; <0.0001) *	NA	

*: statistically significant

Table S1.3. Ward distribution

Ward	2019	2021	2023	Total= 3530
Outpatient	372 (26.25%)	157 (20.52%)	324 (21.76%)	824 (23.34%)
ICU	179 (12.63%)	142 (18.56%)	161 (10.81%)	465 (13.17%)
Pediatrics IV	151 (10.66%)	72 (9.41%)	142 (9.54%)	363 (10.28%)
Surgery	136 (9.60%)	119 (15.56%)	151 (10.14%)	397 (11.25%)
Pediatrics III	85 (6.00%)	24 (3.14%)	140 (9.40%)	248 (7.03%)
Pediatrics I	73 (5.15%)	40 (5.23%)		112 (3.17%)
Nephrology	67 (4.73%)	32 (4.18%)	124 (8.33%)	217 (6.15%)
NICU	66 (4.66%)	42 (5.49%)	45 (3.02%)	153 (4.33%)
Preterm	65 (4.59%)	33 (4.31%)	51 (3.43%)	149 (4.22%)
Oncology	58 (4.09%)	40 (5.23%)	65 (4.37%)	149 (4.22%)
Neonatology	49 (3.46%)	39 (5.10%)	91 (6.11%)	179 (5.07%)
Pediatrics II	43 (3.03%)	9 (1.18%)	40 (2.69%)	91 (2.58%)
Gastroenterology	32 (2.26%)	24 (3.14%)	39 (2.62%)	94 (2.66%)
Other*	31 (2.19%)	17 (2.22%)	43 (2.89%)	90 (2.55%)
ENT	30 (2.12%)	5 (0.65%)	13 (0.87%)	45 (1.27%)
Hematology	29 (2.05%)	9 (1.18%)	22 (1.48%)	59 (1.67%)
Nutritional recovery	23 (1.62%)	7 (0.92%)	13 (0.87%)	41 (1.16%)
Palliative care	22 (1.55%)	19 (2.48%)	26 (1.75%)	66 (1.87%)
Orthopedics	14 (0.99%)	4 (0.52%)	12 (0.81%)	30 (0.85%)
Allergology	13 (0.92%)	6 (0.78%)	31 (2.08%)	48 (1.36%)
AIDS	13 (0.92%)	4 (0.52%)	14 (0.94%)	28 (0.79%)
ED	11 (0.78%)	18 (2.35%)	25 (1.68%)	54 (1.53%)
Cardiology	8 (0.56%)	9 (1.18%)	20 (1.34%)	36 (1.02%)
Pneumology	4 (0.28%)	11 (1.44%)	23 (1.54%)	37 (1.05%)
Dialysis	3 (0.21%)	10 (1.31%)	5 (0.34%)	15 (0.42%)
Infectious diseases			50 (3.36%)	50 (1.42%)

*: Other wards included: Genetics, Neurology, Psychiatry2, Diabetes, Endocrinology, Ophthalmology, Rheumatology, TB=7, Toxicology; AIDS= Acquired Immunodeficiency Syndrome

Table S1.4. Sample distribution

	2019	2021	2023	Total= 6885
Urine	718 (28.92%)	511 (30.56%)	757 (27.73%)	1986 (28.85%)
Nasal secretion	377 (15.18%)	174 (10.41%)	350 (12.82%)	901 (13.09%)
Wound secretion	220 (8.86%)	265 (15.85%)	286 (10.48%)	771 (11.20%)
Hypopharyngeal aspirate	214 (8.62%)	196 (11.72%)	244 (8.94%)	654 (9.50%)
Pharyngeal exudate	201 (8.10%)	19 (1.14%)	337 (12.34%)	557 (8.09%)
Other	173 (6.97%)	21 (1.26%)	28 (1.03%)	222 (3.22%)
Peritoneal fluid	101 (4.07%)	90 (5.38%)	75 (2.75%)	266 (3.86%)
Blood	96 (3.87%)	77 (4.61%)	196 (7.18%)	369 (5.36%)
Catheter	84 (3.38%)	133 (7.95%)	134 (4.91%)	351 (5.10%)
Otic secretion	76 (3.06%)	46 (2.75%)	69 (2.53%)	191 (2.77%)
Conjunctival secretion	48 (1.93%)	34 (2.03%)	87 (3.19%)	169 (2.45%)
Pleural fluid	40 (1.61%)	34 (2.03%)	27 (0.99%)	101 (1.47%)
Umbilical secretion	32 (1.29%)	11 (0.66%)	34 (1.25%)	77 (1.12%)
CSF	30 (1.21%)	11 (0.66%)	14 (0.51%)	55 (0.80%)
Puss	21 (0.85%)	12 (0.72%)	14 (0.51%)	47 (0.68%)
Stool	21 (0.85%)	13 (0.78%)	17 (0.62%)	51 (0.74%)

Skin	18 (0.72%)	11 (0.66%)	15 (0.55%)	44 (0.64%)
Vaginal secretion	12 (0.48%)	4 (0.24%)	11 (0.40%)	27 (0.39%)
Oral lesion	1 (0.04%)	6 (0.36%)	20 (0.73%)	27 (0.39%)
Male genital secretion	-	4 (0.24%)	15 (0.55%)	19 (0.28%)

Table S1.5. Pathogen identification rates

**Pathogen- Total**	**2019**	**2021**	**2023**	**Total= 6885**
Escherichia coli	415 (16.71%)	313 (18.72%)	442 (16.19%)	1170 (16.99%)
Staphylococcus aureus	360 (14.50%)	255 (15.25%)	346 (12.67%)	961 (13.96%)
Klebsiella pneumoniae	229 (9.22%)	138 (8.25%)	227 (8.32%)	594 (8.63%)
Pseudomonas aeruginosa	224 (9.02%)	187 (11.18%)	197 (7.22%)	608 (8.83%)
Streptococcus pneumoniae	199 (8.01%)	72 (4.31%)	186 (6.81%)	457 (6.64%)
Streptococcus group A	191 (7.69%)	14 (0.84%)	381 (13.96%)	586 (8.51%)
Candida albicans	122 (4.91%)	129 (7.72%)	135 (4.95%)	386 (5.61%)
CoNS	119 (4.79%)	70 (4.19%)	190 (6.96%)	379 (5.50%)
Proteus mirabilis	85 (3.42%)	26 (1.56%)	65 (2.38%)	176 (2.56%)
Candida parapsilosis	65 (2.62%)	53 (3.17%)	28 (1.03%)	146 (2.12%)
Enterococcus faecalis	58 (2.34%)	26 (1.56%)	53 (1.94%)	137 (1.99%)
Serratia marcescens	57 (2.30%)	40 (2.39%)	22 (0.81%)	119 (1.73%)
Stenotrophomonas maltophilia	44 (1.77%)	85 (5.08%)	58 (2.12%)	187 (2.72%)
Enterobacter spp.	43 (1.73%)	46 (2.75%)	40 (1.47%)	129 (1.87%)
Candida spp. (other)	36 (1.45%)	37 (2.21%)	27 (0.99%)	100 (1.45%)
Acinetobacter baumannii	34 (1.37%)	22 (1.32%)	39 (1.43%)	95 (1.38%)
Enterococcus faecium	29 (1.17%)	39 (2.33%)	65 (2.38%)	133 (1.93%)
Klebsiella spp. (other)	28 (1.13%)	12 (0.72%)	18 (0.66%)	58 (0.84%)
Other	20 (0.81%)	17 (1.02%)	9 (0.33%)	46 (0.67%)
Sphingomonas paucimobilis	18 (0.72%)	2 (0.12%)	2 (0.07%)	22 (0.32%)
Candida tropicalis	17 (0.68%)	14 (0.84%)	8 (0.29%)	39 (0.57%)
Enterococcus spp. (other)	15 (0.60%)	17 (1.02%)	83 (3.04%)	115 (1.67%)
Citrobacter spp.	14 (0.56%)	7 (0.42%)	13 (0.48%)	34 (0.49%)
Streptococcus spp. (other)	12 (0.48%)	3 (0.18%)	17 (0.62%)	32 (0.46%)
Acinetobacter spp. (other)	10 (0.40%)	8 (0.48%)	15 (0.55%)	33 (0.48%)
Chryseobacterium spp.	9 (0.36%)	14 (0.84%)		23 (0.33%)
Morganella spp.	9 (0.36%)	3 (0.18%)	7 (0.26%)	19 (0.28%)
Proteus spp. (other)	5 (0.20%)	3 (0.18%)	2 (0.07%)	10 (0.15%)
Pseudomonas spp. (other)	5 (0.20%)	5 (0.30%)	15 (0.55%)	25 (0.36%)
Streptococcus group B	5 (0.20%)	5 (0.30%)	11 (0.40%)	21 (0.31%)
Salmonella spp.	4 (0.16%)	4 (0.24%)	15 (0.55%)	23 (0.33%)
Serratia spp. (other)	2 (0.08%)	6 (0.36%)	1 (0.04%)	9 (0.13%)
Haemophilus influenzae			13 (0.48%)	13 (0.19%)

CoNS: Coagulase Negative Staphylococcus
