# Supplementary material for: The Impact of the COVID-19 Pandemic on Pediatric Microbial Resistance Patterns and Abandonment Rates in Western Romania—An Interdisciplinary Study
Source: Antibiotics (Basel). 2025 Apr 16;14(4):411. doi: 10.3390/antibiotics14040411 (PMC12024448; doi:10.3390/antibiotics14040411)
Supplement: Supplementary file 1 [file antibiotics-14-00411-s001.zip › supplement S2- antimicrobials and resistance.pdf]

Table S2.1. Penicillins used for antimicrobial testing

Amoxicillin	2019	2021	2023	Total/ type
Intermediary	0 0 (0.00%)	0 (0.00%)	0 0 (0.00%)	0 0 (0.00%)
Resistant	0 0 (0.00%)	0 (0.00%)	0 0 (0.00%)	0 0 (0.00%)
Susceptible	0 0 (0.00%)	1 (100.00%)	0 0 (0.00%)	1 (100.00%)
Total/ year	0 0 (0.00%)	1 (100.00%)	0 0 (0.00%)	1 (0.02%) *
**Ampicillin**				
Intermediary	8 (0.78%)	4 (0.69%)	5 (0.64%)	17 (0.71%)
Resistant	603 (59.00%)	435 (75.00%)	514 (65.48%)	1552 (65.02%)
Susceptible	411 (40.22%)	141 (24.31%)	266 (33.89%)	818 (34.27%)
Total/ year	1022 (42.82%)	580 (24.30%)	785 (32.89%)	2387 (39.22%) *
**Oxacillin**				
Intermediary	0 0 (0.00%)	0 0 (0.00%)	58 (7.30%)	58 (3.86%)
Resistant	127 (33.51%)	152 (46.20%)	271 (34.13%)	550 (36.62%)
Susceptible	252 (66.49%)	177 (53.80%)	465 (58.56%)	894 (59.52%)
Total/ year	379 (25.23%)	329 (21.90%)	794 (52.86%)	1502 (24.68%) *
**Penicillin**				
Intermediary	4 (1.43%)	0 0 (0.00%)	0 0 (0.00%)	4 (0.47%)
Resistant	114 (40.86%)	49 (48.51%)	160 (34.12%)	323 (38.04%)
Susceptible	161 (57.71%)	52 (51.49%)	309 (65.88%)	522 (61.48%)
Total/ year	279 (32.86%)	101 (11.90%)	469 (55.24%)	849 (13.95%) *
**Piperacillin**				
Intermediary	58 (8.88%)	1 (0.75%)	18 (8.18%)	77 (7.65%)
Resistant	210 (32.16%)	74 (55.64%)	126 (57.27%)	410 (40.76%)
Susceptible	385 (58.96%)	58 (43.61%)	76 (34.55%)	519 (51.59%)
Total/ year	653 (64.91%)	133 (13.22%)	220 (21.87%)	1006 (16.53%) *
**Methicillin**				
Intermediary	25 (2.49%)	0 0 (0.00%)	0 0 (0.00%)	25 (1.58%)
Resistant	212 (21.07%)	60 (32.61%)	64 (16.49%)	336 (21.29%)
Susceptible	769 (76.44%)	124 (67.39%)	324 (83.51%)	1217 (77.12%)
Total/ year	1006 (63.75%)	184 (11.66%)	388 (24.59%)	1578 (25.93%) *
**Ticarcillin**				
Intermediary	8 (4.28%)	1 (0.83%)	19 (2.29%)	28 (2.46%)
Resistant	123 (65.78%)	62 (51.67%)	304 (36.67%)	489 (43.05%)
Susceptible	56 (29.95%)	57 (47.50%)	506 (61.04%)	619 (54.49%)
Total/ year	187 (16.46%)	120 (10.56%)	829 (72.98%)	1136 (18.67%) *
**Penicillin class total**				
Intermediary	99 (3.98%)	6 (0.54%)	100 (4.00%)	205 (3.37%)
Resistant	1037 (41.73%)	683 (61.92%)	1125 (45.04%)	2845 (46.75%)
Susceptible	1349 (54.29%)	414 (37.53%)	1273 (50.96%)	3036 (49.88%)
Total/ year	2485 (40.83%)	1103 (18.12%)	2498 (41.05%)	6086 (88.40%%) **

*: reported to class total; **: reported tot total number of samples, n= 6885)

Table S2.2. Cephalosporins used for antimicrobial testing

Cefepime	2019	2021	2023	Total/ type
Intermediary	0 (0.00%)	0 (0.00%)	138 (17.97%)	138 (16.55%)
Resistant	30 (69.77%)	16 (69.57%)	228 (29.69%)	274 (32.85%)
Susceptible	13 (30.23%)	7 (30.43%)	402 (52.34%)	422 (50.60%)
Total/ year	43 (5.16%)	23 (2.76%)	768 (92.09%)	834 (17.01%) *

**Cefotaxime**				
Intermediary	5 (0.62%)	3 (0.63%)	81 (10.98%)	89 (4.38%)
Resistant	350 (43.10%)	210 (43.75%)	243 (32.93%)	803 (39.56%)
Susceptible	457 (56.28%)	267 (55.63%)	414 (56.10%)	1138 (56.06%)
Total/ year	812 (40.00%)	480 (23.65%)	738 (36.35%)	2030 (41.40%) *
**Cefoxitin**				
Intermediary	0 (0.00%)	0 (0.00%)	0 (0.00%)	0 (0.00%)
Resistant	178 (39.64%)	97 (55.11%)	93 (18.60%)	368 (32.71%)
Susceptible	271 (60.36%)	79 (44.89%)	407 (81.40%)	757 (67.29%)
Total/ year	449 (39.91%)	176 (15.64%)	500 (44.44%)	1125 (22.95%) *
**Ceftazidime**				
Intermediary	48 (4.11%)	24 (2.83%)	60 (10.99%)	132 (5.15%)
Resistant	435 (37.21%)	314 (37.07%)	160 (29.30%)	909 (35.48%)
Susceptible	686 (58.68%)	509 (60.09%)	326 (59.71%)	1521 (59.37%)
Total/ year	1169 (45.63%)	847 (33.06%)	546 (21.31%)	2562 (52.25%) *
**Ceftibuten**				
Intermediary	0 (0.00%)	3 (15.00%)	98 (51.31%)	101 (39.45%)
Resistant	2 (4.44%)	4 (20.00%)	67 (35.08%)	73 (28.52%)
Susceptible	43 (95.56%)	13 (65.00%)	26 (13.61%)	82 (32.03%)
Total/ year	45 (17.58%)	20 (7.81%)	191 (74.61%)	256 (5.22%) *
**Ceftizoxime**				
Intermediary	0 (0.00%)	0 0 (0.00%)	0 0 (0.00%)	0 (0.00%)
Resistant	204 (36.36%)	0 0 (0.00%)	0 0 (0.00%)	204 (36.36%)
Susceptible	357 (63.64%)	0 0 (0.00%)	0 0 (0.00%)	357 (63.64%)
Total/ year	561 (100.00%)	0 0 (0.00%)	0 0 (0.00%)	561 (11.44%) *
**Ceftriaxone**				
Intermediary	0 (0.00%)	0 (0.00%)	0 (0.00%)	0 (0.00%)
Resistant	151 (32.75%)	138 (29.68%)	44 (12.72%)	333 (26.18%)
Susceptible	310 (67.25%)	327 (70.32%)	302 (87.28%)	939 (73.82%)
Total/ year	461 (36.24%)	465 (36.56%)	346 (27.20%)	1272 (25.94%) *
**Cefuroxime**				
Intermediary	0 0 (0.00%)	0 0 (0.00%)	221 (29.78%)	221 (29.78%)
Resistant	0 0 (0.00%)	0 0 (0.00%)	238 (32.08%)	238 (32.08%)
Susceptible	0 0 (0.00%)	0 0 (0.00%)	283 (38.14%)	283 (38.14%)
Total/ year	0 0 (0.00%)	0 0 (0.00%)	742 (100.00%)	742 (15.13%) *
**Cephalosporin class total**				
Intermediary	57 (2.94%)	35 (3.05%)	277 (15.23%)	369 (7.53%)
Resistant	675 (34.87%)	481 (41.90%)	582 (32.00%)	1738 (35.45%)
Susceptible	1204 (62.19%)	632 (55.05%)	960 (52.78%)	2796 (57.03%)
Total/ year	1936 (39.49%)	1148 (23.41%)	1819 (37.10%)	4903 (71.21%) **

*: reported to class total; **: reported tot total number of samples, n= 6885)

Table S2.3. Combination therapy (penicillins and cephalosporins) used for antimicrobial testing

**Amoxicillin/ Clavulanic Acid**	**2019**	**2021**	**2023**	**Total/ type**
Intermediary	134 (11.86%)	58 (7.47%)	3 (0.30%)	195 (6.72%)
Resistant	502 (44.42%)	351 (45.23%)	524 (52.66%)	1377 (47.47%)
Susceptible	494 (43.72%)	367 (47.29%)	468 (47.04%)	1329 (45.81%)
Total/ year	1130 (38.95%)	776 (26.75%)	995 (34.30%)	2901 (61.53%) *
**Ampicillin/ Sulbactam**				

Intermediary	2 (1.54%)	1 (1.35%)	1 (0.83%)	4 (1.23%)
Resistant	76 (58.46%)	44 (59.46%)	47 (39.17%)	167 (51.54%)
Susceptible	52 (40.00%)	29 (39.19%)	72 (60.00%)	153 (47.22%)
Total/ year	130 (40.12%)	74 (22.84%)	120 (37.04%)	324 (6.87%) *
**Ceftazidime/ Avibactam**				
Intermediary	0 0 (0.00%)	0 (0.00%)	1 (0.17%)	1 (0.17%)
Resistant	0 0 (0.00%)	0 (0.00%)	65 (10.91%)	65 (10.83%)
Susceptible	0 0 (0.00%)	4 (100.00%)	530 (88.93%)	534 (89.00%)
Total/ year	0 0 (0.00%)	4 (0.67%)	596 (99.33%)	600 (12.73%) *
**Piperacillin/ Tazobactam**				
Intermediary	47 (8.29%)	38 (5.23%)	39 (5.55%)	124 (6.21%)
Resistant	123 (21.69%)	155 (21.35%)	189 (26.88%)	467 (23.40%)
Susceptible	397 (70.02%)	533 (73.42%)	475 (67.57%)	1405 (70.39%)
Total/ year	567 (28.41%)	726 (36.37%)	703 (35.22%)	1996 (42.33%) *
**Ticarcillin/ Clavulanic Acid**				
Intermediary	27 (10.00%)	24 (18.60%)	19 (3.23%)	70 (7.09%)
Resistant	72 (26.67%)	58 (44.96%)	119 (20.24%)	249 (25.23%)
Susceptible	171 (63.33%)	47 (36.43%)	450 (76.53%)	668 (67.68%)
Total/ year	270 (27.36%)	129 (13.07%)	588 (59.57%)	987 (20.93%) *
**Combination therapies class total**				
Intermediary	202 (12.83%)	113 (9.31%)	49 (2.54%)	364 (7.72%)
Resistant	609 (38.67%)	459 (37.81%)	659 (34.22%)	1727 (36.63%)
Susceptible	764 (48.51%)	642 (52.88%)	1218 (63.24%)	2624 (55.65%)
Total/ year	1575 (33.40%)	1214 (25.75%)	1926 (40.85%)	4715 (68.48%) **

*: reported to class total; **: reported tot total number of samples, n= 6885)

Table S2.4. Carbapenems used for antimicrobial testing

**Ertapenem**	**2019**	**2021**	**2023**	**Total/ type**
Intermediary	10 (1.48%)	8 (1.62%)	0 (0.00%)	18 (1.16%)
Resistant	173 (25.63%)	19 (3.85%)	24 (6.35%)	216 (13.96%)
Susceptible	492 (72.89%)	467 (94.53%)	354 (93.65%)	1313 (84.87%)
Total/ year	675 (43.63%)	494 (31.93%)	378 (24.43%)	1547 (51.77%) *
**Imipenem**				
Intermediary	40 (7.29%)	25 (3.55%)	121 (18.03%)	186 (9.67%)
Resistant	124 (22.59%)	129 (18.32%)	104 (15.50%)	357 (18.56%)
Susceptible	385 (70.13%)	550 (78.13%)	446 (66.47%)	1381 (71.78%)
Total/ year	549 (28.53%)	704 (36.59%)	671 (34.88%)	1924 (64.39%) *
**Meropenem**				
Intermediary	21 (3.13%)	15 (1.88%)	8 (0.89%)	44 (1.86%)
Resistant	98 (14.58%)	130 (16.33%)	147 (16.42%)	375 (15.87%)
Susceptible	553 (82.29%)	651 (81.78%)	740 (82.68%)	1944 (82.27%)
Total/ year	672 (28.44%)	796 (33.69%)	895 (37.88%)	2363 (79.08%) *
**Carbapenem class total**				
Intermediary	61 (5.84%)	45 (5.21%)	127 (11.77%)	233 (7.80%)
Resistant	277 (26.51%)	152 (17.59%)	190 (17.61%)	619 (20.72%)
Susceptible	707 (67.66%)	667 (77.20%)	762 (70.62%)	2136 (71.49%)
Total/ year	1045 (34.97%)	864 (28.92%)	1079 (36.11%)	2988 (43.40%) **

*: reported to class total; **: reported tot total number of samples, n= 6885)

Table S2.5. Fluoroquinolones used for antimicrobial testing

**Ciprofloxacin**	**2019**	**2021**	**2023**	**Total/ type**
Intermediary	26 (1.47%)	38 (3.09%)	180 (10.59%)	244 (5.20%)
Resistant	537 (30.39%)	385 (31.33%)	504 (29.66%)	1426 (30.37%)
Susceptible	1204 (68.14%)	806 (65.58%)	1015 (59.74%)	3025 (64.43%)
Total/ year	1767 (37.64%)	1229 (26.18%)	1699 (36.19%)	4695 (73.27%) *
**Levofloxacin**				
Intermediary	0 (0.00%)	0 (0.00%)	91 (17.67%)	91 (4.40%)
Resistant	211 (24.94%)	152 (21.44%)	171 (33.20%)	534 (25.80%)
Susceptible	635 (75.06%)	557 (78.56%)	253 (49.13%)	1445 (69.81%)
Total/ year	846 (40.87%)	709 (34.25%)	515 (24.88%)	2070 (32.30%) *
**Moxifloxacin**				
Intermediary	18 (4.30%)	17 (6.85%)	0 (0.00%)	35 (3.65%)
Resistant	46 (10.98%)	33 (13.31%)	21 (7.17%)	100 (10.42%)
Susceptible	355 (84.73%)	198 (79.84%)	272 (92.83%)	825 (85.94%)
Total/ year	419 (43.65%)	248 (25.83%)	293 (30.52%)	960 (14.98%) *
**Norfloxacin**				
Intermediary	4 (0.52%)	23 (4.82%)	0 (0.00%)	27 (1.32%)
Resistant	311 (40.76%)	148 (31.03%)	396 (49.13%)	855 (41.79%)
Susceptible	448 (58.72%)	306 (64.15%)	410 (50.87%)	1164 (56.89%)
Total/ year	763 (37.29%)	477 (23.31%)	806 (39.39%)	2046 (31.93%) *
**Ofloxacin**				
Intermediary	0 (0.00%)	0 0 (0.00%)	29 (14.80%)	29 (6.43%)
Resistant	81 (31.76%)	0 0 (0.00%)	126 (64.29%)	207 (45.90%)
Susceptible	174 (68.24%)	0 0 (0.00%)	41 (20.92%)	215 (47.67%)
Total/ year	255 (56.54%)	0 0 (0.00%)	196 (43.46%)	451 (7.04%) *
**Fluoroquinolone class total**				
Intermediary	40 (1.71%)	75 (5.19%)	265 (10.09%)	380 (5.93%)
Resistant	665 (28.47%)	398 (27.54%)	848 (32.28%)	1911 (29.82%)
Susceptible	1631 (69.82%)	972 (67.27%)	1514 (57.63%)	4117 (64.25%)
Total/ year	2336 (36.45%)	1445 (22.55%)	2627 (41.00%)	6408 (93.07%) **

*: reported to class total; **: reported tot total number of samples, n= 6885)

Table S2.6. Aminoglycosides used for antimicrobial testing

**Amikacin**	**2019**	**2021**	**2023**	**Total/ type**
Intermediary	62 (5.49%)	40 (4.91%)	9 (0.82%)	111 (3.64%)
Resistant	133 (11.77%)	108 (13.25%)	144 (13.06%)	385 (12.63%)
Susceptible	935 (82.74%)	667 (81.84%)	950 (86.13%)	2552 (83.73%)
Total/ year	1130 (37.07%)	815 (26.74%)	1103 (36.19%)	3048 (49.17%) *
**Gentamycin**				
Intermediary	6 (0.75%)	8 (0.69%)	138 (9.81%)	152 (4.50%)
Resistant	249 (31.05%)	321 (27.53%)	343 (24.38%)	913 (27.05%)
Susceptible	547 (68.20%)	837 (71.78%)	926 (65.81%)	2310 (68.44%)
Total/ year	802 (23.76%)	1166 (34.55%)	1407 (41.69%)	3375 (54.44%) *
**Gentamycin HL**				
Intermediary	5 (0.50%)	0 (0.00%)	0 (0.00%)	5 (0.44%)
Resistant	292 (29.20%)	47 (66.20%)	29 (40.28%)	368 (32.20%)
Susceptible	703 (70.30%)	24 (33.80%)	43 (59.72%)	770 (67.37%)
Total/ year	1000 (87.49%)	71 (6.21%)	72 (6.30%)	1143 (18.44%) *

**Kanamycin**				
Intermediary	0 (0.00%)	4 (2.58%)	0 (0.00%)	4 (0.60%)
Resistant	23 (15.13%)	32 (20.65%)	24 (6.74%)	79 (11.92%)
Susceptible	129 (84.87%)	119 (76.77%)	332 (93.26%)	580 (87.48%)
Total/ year	152 (22.93%)	155 (23.38%)	356 (53.70%)	663 (10.70%) *
**Neomycin**				
Intermediary	0 0 (0.00%)	0 0 (0.00%)	1 (0.57%)	1 (0.57%)
Resistant	0 0 (0.00%)	0 0 (0.00%)	42 (24.14%)	42 (24.14%)
Susceptible	0 0 (0.00%)	0 0 (0.00%)	131 (75.29%)	131 (75.29%)
Total/ year	0 0 (0.00%)	0 0 (0.00%)	174 (100.00%)	174 (2.81%) *
**Netilmicin**				
Intermediary	1 (0.16%)	2 (0.46%)	0 (0.00%)	3 (0.22%)
Resistant	155 (24.37%)	120 (27.52%)	86 (28.96%)	361 (26.37%)
Susceptible	480 (75.47%)	314 (72.02%)	211 (71.04%)	1005 (73.41%)
Total/ year	636 (46.46%)	436 (31.85%)	297 (21.69%)	1369 (22.08%) *
**Streptomycin- HL**				
Intermediary	2 (1.32%)	0 (0.00%)	40 (18.43%)	42 (11.35%)
Resistant	9 (5.92%)	0 (0.00%)	111 (51.15%)	120 (32.43%)
Susceptible	141 (92.76%)	1 (100.00%)	66 (30.41%)	208 (56.22%)
Total/ year	152 (41.08%)	1 (0.27%)	217 (58.65%)	370 (5.97%) *
**Tobramycin**				
Intermediary	11 (1.22%)	3 (0.97%)	0 (0.00%)	14 (0.93%)
Resistant	308 (34.18%)	65 (21.04%)	97 (33.45%)	470 (31.33%)
Susceptible	582 (64.59%)	241 (77.99%)	193 (66.55%)	1016 (67.73%)
Total/ year	901 (60.07%)	309 (20.60%)	290 (19.33%)	1500 (24.20%) *
**Aminoglycoside class total**				
Intermediary	85 (3.55%)	56 (3.71%)	173 (7.55%)	314 (5.07%)
Resistant	744 (31.04%)	424 (28.08%)	563 (24.56%)	1731 (27.92%)
Susceptible	1568 (65.42%)	1030 (68.21%)	1556 (67.89%)	4154 (67.01%)
Total/ year	2397 (38.67%)	1510 (24.36%)	2292 (36.97%)	6199 (90.04%) **

*: reported to class total; **: reported tot total number of samples, n= 6885)

Table S2.7. Macrolides used for antimicrobial testing

Azithromycin	2019	2021	2023	Total/ type
Intermediary	0 (0.00%)	0 (0.00%)	63 (19.63%)	63 (12.28%)
Resistant	133 (69.63%)	0 (0.00%)	133 (41.43%)	266 (51.85%)
Susceptible	58 (30.37%)	1 (100.00%)	125 (38.94%)	184 (35.87%)
Total/ year	191 (37.23%)	1 (0.19%)	321 (62.57%)	513 (28.14%) *
**Clarithromycin**				
Intermediary	0 0 (0.00%)	0 (0.00%)	0 0 (0.00%)	0 (0.00%)
Resistant	0 0 (0.00%)	0 (0.00%)	0 0 (0.00%)	0 (0.00%)
Susceptible	0 0 (0.00%)	1 (100.00%)	0 0 (0.00%)	1 (100.00%)
Total/ year	0 0 (0.00%)	1 (100.00%)	0 0 (0.00%)	1 (0.05%) *
**Erythromycin**				
Intermediary	5 (0.93%)	7 (2.06%)	2 (0.36%)	14 (0.98%)
Resistant	251 (46.92%)	224 (66.08%)	183 (33.39%)	658 (46.27%)
Susceptible	279 (52.15%)	108 (31.86%)	363 (66.24%)	750 (52.74%)
Total/ year	535 (37.62%)	339 (23.84%)	548 (38.54%)	1422 (78.00%) *
**Macrolide class total**				

Intermediary	5 (0.77%)	7 (2.06%)	65 (7.76%)	77 (4.22%)
Resistant	321 (49.69%)	224 (66.08%)	298 (35.56%)	843 (46.24%)
Susceptible	320 (49.54%)	108 (31.86%)	475 (56.68%)	903 (49.53%)
Total/ year	646 (35.44%)	339 (18.60%)	838 (45.97%)	1823 (26.48%) **

*: reported to class total; **: reported tot total number of samples, n= 6885)

Table S2.8. Lincosamides used for antimicrobial testing

**Clindamycin**	**2019**	**2021**	**2023**	**Total/ type**
Intermediary	3 (0.73%)	0 (0.00%)	0 (0.00%)	3 (0.24%)
Resistant	162 (39.51%)	133 (49.44%)	122 (21.22%)	417 (33.25%)
Susceptible	245 (59.76%)	136 (50.56%)	453 (78.78%)	834 (66.51%)
Total/ year	410 (32.70%)	269 (21.45%)	575 (45.85%)	1254 (80.33%) *
**Clindamycin- inducible**				
Intermediary	0 (0.00%)	0 (0.00%)	0 (0.00%)	0 (0.00%)
Resistant	114 (42.70%)	34 (29.57%)	52 (34.44%)	200 (37.52%)
Susceptible	153 (57.30%)	81 (70.43%)	99 (65.56%)	333 (62.48%)
Total/ year	267 (50.09%)	115 (21.58%)	151 (28.33%)	533 (34.14%) *
**Lincosamide class total**				
Intermediary	3 (0.49%)	0 (0.00%)	0 (0.00%)	3 (0.19%)
Resistant	236 (38.31%)	134 (45.89%)	160 (24.50%)	530 (33.95%)
Susceptible	377 (61.20%)	158 (54.11%)	493 (75.50%)	1028 (65.86%)
Total/ year	616 (39.46%)	292 (18.71%)	653 (41.83%)	1561 (26.48%) **

*: reported to class total; **: reported tot total number of samples, n= 6885)

Table S2.9. Cyclines used for antimicrobial testing

**Minocycline**	**2019**	**2021**	**2023**	**Total/ type**
Intermediary	11 (22.00%)	6 (30.00%)	0 0 (0.00%)	17 (24.29%)
Resistant	14 (28.00%)	4 (20.00%)	0 0 (0.00%)	18 (25.71%)
Susceptible	25 (50.00%)	10 (50.00%)	0 0 (0.00%)	35 (50.00%)
Total/ year	50 (71.43%)	20 (28.57%)	0 0 (0.00%)	70 (3.67%) *
**Tetracycline**				
Intermediary	1 (0.27%)	1 (0.38%)	3 (0.42%)	5 (0.37%)
Resistant	170 (45.70%)	151 (57.85%)	178 (25.04%)	499 (37.13%)
Susceptible	201 (54.03%)	109 (41.76%)	530 (74.54%)	840 (62.50%)
Total/ year	372 (27.68%)	261 (19.42%)	711 (52.90%)	1344 (70.48%) *
**Tigecycline**				
Intermediary	4 (0.95%)	0 (0.00%)	1 (0.70%)	5 (0.66%)
Resistant	46 (10.95%)	0 (0.00%)	3 (2.11%)	49 (6.46%)
Susceptible	370 (88.10%)	197 (100.00%)	138 (97.18%)	705 (92.89%)
Total/ year	420 (55.34%)	197 (25.96%)	142 (18.71%)	759 (39.80%) *
**Cycline class total**				
Intermediary	14 (1.96%)	7 (1.76%)	4 (0.50%)	25 (1.31%)
Resistant	198 (27.73%)	155 (38.94%)	179 (22.52%)	532 (27.90%)
Susceptible	502 (70.31%)	236 (59.30%)	612 (76.98%)	1350 (70.79%)
Total/ year	714 (37.44%)	398 (20.87%)	795 (41.69%)	1907 (27.70%) **

*: reported to class total; **: reported tot total number of samples, n= 6885)

Table S2.10. Glycopeptides used for antimicrobial testing

Teicoplanin	2019	2021	2023	Total/ type
Intermediary	4 (0.97%)	0 (0.00%)	0 (0.00%)	4 (0.40%)
Resistant	109 (26.39%)	7 (3.50%)	18 (4.71%)	134 (13.47%)
Susceptible	300 (72.64%)	193 (96.50%)	364 (95.29%)	857 (86.13%)
Total/ year	413 (41.51%)	200 (20.10%)	382 (38.39%)	995 (71.79%) *
**Vancomycin**				
Intermediary	1 (0.28%)	0 (0.00%)	1 (0.34%)	2 (0.21%)
Resistant	7 (1.96%)	6 (2.10%)	10 (3.39%)	23 (2.45%)
Susceptible	349 (97.76%)	280 (97.90%)	284 (96.27%)	913 (97.33%)
Total/ year	357 (38.06%)	286 (30.49%)	295 (31.45%)	938 (67.68%) *
**Glycopeptide class total**				
Intermediary	5 (0.84%)	0 (0.00%)	1 (0.20%)	6 (0.43%)
Resistant	113 (18.99%)	9 (3.10%)	20 (3.99%)	142 (10.25%)
Susceptible	482 (81.01%)	281 (96.90%)	481 (96.01%)	1244 (89.75%)
Total/ year	595 (42.93%)	290 (20.92%)	501 (36.15%)	1386 (20.13%) **

*: reported to class total; **: reported tot total number of samples, n= 6885)

Table S2.11. Urinary antibiotics used for antimicrobial testing

Nalidixic Acid	2019	2021	2023	Total/ type
Intermediary	1 (1.79%)	0 (0.00%)	0 (0.00%)	1 (1.02%)
Resistant	16 (28.57%)	6 (31.58%)	5 (21.74%)	27 (27.55%)
Susceptible	39 (69.64%)	13 (68.42%)	18 (78.26%)	70 (71.43%)
Total/ year	56 (57.14%)	19 (19.39%)	23 (23.47%)	98 (3.25%) *
**Fosfomycin**				
Intermediary	7 (1.37%)	9 (2.17%)	2 (0.17%)	18 (0.87%)
Resistant	35 (6.84%)	34 (8.19%)	388 (33.77%)	457 (22.01%)
Susceptible	470 (91.80%)	372 (89.64%)	759 (66.06%)	1601 (77.12%)
Total/ year	512 (24.66%)	415 (19.99%)	1149 (55.35%)	2076 (68.76%) *
**Nitrofurantoin**				
Intermediary	108 (14.06%)	55 (12.59%)	0 (0.00%)	163 (9.44%)
Resistant	194 (25.26%)	90 (20.59%)	163 (31.29%)	447 (25.90%)
Susceptible	466 (60.68%)	292 (66.82%)	358 (68.71%)	1116 (64.66%)
Total/ year	768 (44.50%)	437 (25.32%)	521 (30.19%)	1726 (57.17%) *
**Urinary antibiotic class total**				
Intermediary	113 (11.73%)	63 (10.29%)	2 (0.14%)	178 (5.90%)
Resistant	235 (24.40%)	117 (19.12%)	479 (33.17%)	831 (27.53%)
Susceptible	728 (75.60%)	495 (80.88%)	965 (66.83%)	2188 (72.47%)
Total/ year	963 (31.90%)	612 (20.27%)	1444 (47.83%)	3019 (43.85%) **

*: reported to class total; **: reported tot total number of samples, n= 6885)

Table S2.12. Reserve antibiotics used for antimicrobial testing

Colistin	2019	2021	2023	Total/ type
Intermediary	1 (0.53%)	61 (29.76%)	2 (0.23%)	64 (5.10%)
Resistant	30 (15.87%)	37 (18.05%)	329 (38.26%)	396 (31.58%)
Susceptible	158 (83.60%)	107 (52.20%)	529 (61.51%)	794 (63.32%)
Total/ year	189 (15.07%)	205 (16.35%)	860 (68.58%)	1254 (25.82%) *

**Linezolid**				
Intermediary	0 (0.00%)	1 (0.38%)	11 (2.24%)	12 (0.72%)
Resistant	206 (22.49%)	3 (1.15%)	36 (7.35%)	245 (14.69%)
Susceptible	710 (77.51%)	258 (98.47%)	443 (90.41%)	1411 (84.59%)
Total/ year	916 (54.92%)	262 (15.71%)	490 (29.38%)	1668 (34.35%) *
**Trimethoprim/ Sulfamethoxazole**				
Intermediary	1 (0.11%)	0 (0.00%)	14 (1.92%)	15 (0.57%)
Resistant	302 (32.20%)	400 (42.37%)	271 (37.12%)	973 (37.25%)
Susceptible	635 (67.70%)	544 (57.63%)	445 (60.96%)	1624 (62.17%)
Total/ year	938 (35.91%)	944 (36.14%)	730 (27.95%)	2612 (53.79%) *
**Reserve antibiotic class total**				
Intermediary	2 (0.11%)	62 (5.22%)	27 (1.46%)	91 (1.87%)
Resistant	528 (28.98%)	436 (36.73%)	604 (32.70%)	1568 (32.29%)
Susceptible	1294 (71.02%)	751 (63.27%)	1243 (67.30%)	3288 (67.71%)
Total/ year	1822 (37.52%)	1187 (24.44%)	1847 (38.04%)	4856 (70.53%) **

*: reported to class total; **: reported tot total number of samples, n= 6885)

Table S2.13. Other antibiotics used for antimicrobial testing

**Aztreonam**	**2019**	**2021**	**2023**	**Total/ type**
Intermediary	0 (0.00%)	0 (0.00%)	138 (17.97%)	138 (16.55%)
Resistant	30 (69.77%)	16 (69.57%)	228 (29.69%)	274 (32.85%)
Susceptible	13 (30.23%)	7 (30.43%)	402 (52.34%)	422 (50.60%)
Total/ year	43 (5.16%)	23 (2.76%)	768 (92.09%)	834 (26.02%) *
**Chloramphenicol**				
Intermediary	0 (0.00%)	0 (0.00%)	0 (0.00%)	0 (0.00%)
Resistant	18 (3.77%)	15 (7.11%)	30 (18.07%)	63 (7.37%)
Susceptible	460 (96.23%)	196 (92.89%)	136 (81.93%)	792 (92.63%)
Total/ year	478 (55.91%)	211 (24.68%)	166 (19.42%)	855 (26.68%) *
**Fusidic acid**				
Intermediary	15 (2.79%)	0 (0.00%)	5 (1.81%)	20 (2.08%)
Resistant	73 (13.59%)	35 (23.49%)	109 (39.35%)	217 (22.53%)
Susceptible	449 (83.61%)	114 (76.51%)	163 (58.84%)	726 (75.39%)
Total/ year	537 (55.76%)	149 (15.47%)	277 (28.76%)	963 (30.05%) *
**Rifampicin**				
Intermediary	2 (0.55%)	0 (0.00%)	8 (1.05%)	10 (0.67%)
Resistant	30 (8.31%)	24 (6.33%)	154 (20.24%)	208 (13.86%)
Susceptible	329 (91.14%)	355 (93.67%)	599 (78.71%)	1283 (85.48%)
Total/ year	361 (24.05%)	379 (25.25%)	761 (50.70%)	1501 (46.83%) *
**Other antibiotics class total**				
Intermediary	17 (1.53%)	0 (0.00%)	151 (9.36%)	168 (5.24%)
Resistant	137 (12.36%)	85 (17.56%)	493 (30.56%)	715 (22.31%)
Susceptible	971 (87.64%)	399 (82.44%)	1120 (69.44%)	2490 (77.69%)
Total/ year	1108 (34.57%)	484 (15.10%)	1613 (50.33%)	3205 (46.55%) **

*: reported to class total; **: reported tot total number of samples, n= 6885)

Table S2.14. Polyenes used for antimicrobial testing

**Amphotericin B**	**2019**	**2021**	**2023**	**Total/ type**
Intermediary	2 (0.83%)	0 (0.00%)	0 (0.00%)	2 (0.29%)

Resistant	8 (3.32%)	3 (1.25%)	10 (5.05%)	21 (3.09%)
Susceptible	231 (95.85%)	237 (98.75%)	188 (94.95%)	656 (96.61%)
Total/ year	241 (35.49%)	240 (35.35%)	198 (29.16%)	679 (64.30%) *
**Nystatin**				
Intermediary	0 (0.00%)	0 (0.00%)	0 (0.00%)	0 (0.00%)
Resistant	14 (5.83%)	1 (0.42%)	123 (27.64%)	138 (14.94%)
Susceptible	226 (94.17%)	238 (99.58%)	322 (72.36%)	786 (85.06%)
Total/ year	240 (25.97%)	239 (25.87%)	445 (48.16%)	924 (87.50%) *
**Polyene class total**				
Intermediary	2 (0.78%)	0 (0.00%)	0 (0.00%)	2 (0.19%)
Resistant	19 (7.36%)	3 (1.24%)	133 (23.92%)	155 (14.68%)
Susceptible	237 (91.86%)	239 (98.76%)	423 (76.08%)	899 (85.13%)
Total/ year	258 (24.43%)	242 (22.92%)	556 (52.65%)	1056 (15.34%) **

*: reported to class total; **: reported tot total number of samples, n= 6885)

Table S2.15. Azoles used for antimicrobial testing

Clotrimazole	2019	2021	2023	Total/ type
Intermediary	4 (1.11%)	0 0 (0.00%)	0 0 (0.00%)	4 (1.11%)
Resistant	120 (33.24%)	0 0 (0.00%)	0 0 (0.00%)	120 (33.24%)
Susceptible	237 (65.65%)	0 0 (0.00%)	0 0 (0.00%)	237 (65.65%)
Total/ year	361 (100.00%)	0 0 (0.00%)	0 0 (0.00%)	361 (13.28%) *
**Econazole**				
Intermediary	4 (2.08%)	0 (0.00%)	0 (0.00%)	4 (0.50%)
Resistant	53 (27.60%)	1 (0.42%)	32 (8.63%)	86 (10.71%)
Susceptible	135 (70.31%)	239 (99.58%)	339 (91.37%)	713 (88.79%)
Total/ year	192 (23.91%)	240 (29.89%)	371 (46.20%)	803 (29.54%) *
**Fluconazole**				
Intermediary	0 (0.00%)	1 (0.42%)	0 (0.00%)	1 (0.17%)
Resistant	19 (7.88%)	30 (12.50%)	20 (16.67%)	69 (11.48%)
Susceptible	222 (92.12%)	209 (87.08%)	100 (83.33%)	531 (88.35%)
Total/ year	241 (40.10%)	240 (39.93%)	120 (19.97%)	601 (22.11%) *
**Itraconazole**				
Intermediary	30 (4.95%)	0 (0.00%)	214 (50.71%)	244 (23.71%)
Resistant	111 (18.32%)	0 (0.00%)	121 (28.67%)	232 (22.55%)
Susceptible	465 (76.73%)	1 (100.00%)	87 (20.62%)	553 (53.74%)
Total/ year	606 (58.89%)	1 (0.10%)	422 (41.01%)	1029 (37.86%) *
**Ketoconazole**				
Intermediary	0 (0.00%)	2 (0.83%)	5 (1.24%)	7 (0.79%)
Resistant	33 (13.64%)	11 (4.58%)	50 (12.41%)	94 (10.62%)
Susceptible	209 (86.36%)	227 (94.58%)	348 (86.35%)	784 (88.59%)
Total/ year	242 (27.34%)	240 (27.12%)	403 (45.54%)	885 (32.56%) *
**Miconazole**				
Intermediary	3 (1.28%)	0 (0.00%)	0 (0.00%)	3 (0.36%)
Resistant	36 (15.32%)	30 (12.50%)	165 (44.84%)	231 (27.40%)
Susceptible	196 (83.40%)	210 (87.50%)	203 (55.16%)	609 (72.24%)
Total/ year	235 (27.88%)	240 (28.47%)	368 (43.65%)	843 (31.02%) *
**Voriconazole**				
Intermediary	0 (0.00%)	0 0 (0.00%)	0 0 (0.00%)	0 (0.00%)
Resistant	5 (6.94%)	0 0 (0.00%)	0 0 (0.00%)	5 (6.94%)

Susceptible	67 (93.06%)	0 0 (0.00%)	0 0 (0.00%)	67 (93.06%)
Total/ year	72 (100.00%)	0 0 (0.00%)	0 0 (0.00%)	72 (2.65%) *
**Azole class total**				
Intermediary	35 (2.65%)	3 (1.07%)	219 (19.62%)	257 (9.46%)
Resistant	314 (23.77%)	39 (13.88%)	318 (28.49%)	671 (24.69%)
Susceptible	972 (73.58%)	239 (85.05%)	579 (51.88%)	1790 (65.86%)
Total/ year	1321 (48.60%)	281 (10.34%)	1116 (41.06%)	2718 (39.48%) **

*: reported to class total; **: reported tot total number of samples, n= 6885)

Table S2.16. Other antifungals used for antimicrobial testing

Caspofungin	2019	2021	2023	Total/ type
Intermediary	0 (0.00%)	0 0 (0.00%)	0 (0.00%)	0 (0.00%)
Resistant	0 (0.00%)	0 0 (0.00%)	27 (28.13%)	27 (27.55%)
Susceptible	2 (100.00%)	0 0 (0.00%)	69 (71.88%)	71 (72.45%)
Total/ year	2 (2.04%)	0 0 (0.00%)	96 (97.96%)	98 (8.89%) *
**Flucytosine**				
Intermediary	0 (0.00%)	0 (0.00%)	1 (0.24%)	1 (0.10%)
Resistant	97 (27.17%)	7 (2.93%)	116 (28.02%)	220 (21.78%)
Susceptible	260 (72.83%)	232 (97.07%)	297 (71.74%)	789 (78.12%)
Total/ year	357 (35.35%)	239 (23.66%)	414 (40.99%)	1010 (91.65%) *
**Other antifungal class total**				
Intermediary	0 (0.00%)	0 (0.00%)	1 0 (0.00%)	1 0 (0.00%)
Resistant	97 0 (0.00%)	7 0 (0.00%)	138 0 (0.00%)	242 0 (0.00%)
Susceptible	261 0 (0.00%)	232 0 (0.00%)	366 0 (0.00%)	859 0 (0.00%)
Total/ year	358 (32.49%)	239 (21.69%)	505 (45.83%)	1102 (16.01%) **

*: reported to class total; **: reported tot total number of samples, n= 6885)

Table S2.17. Incidence of resistance for antimicrobial classes.

Year	2019	2021	2023	Total resistant
Overall	1805 (72.69%)	1121 (67.05%)	1888 (69.16%)	4814 (69.92%)
Penicillins	1037 (57.45%)	683 (60.93%)	1125 (59.59%)	2845 (59.10%)
Aminoglicosides	675 (41.22%)	481 (37.82%)	582 (29.82%)	1738 (35.96%)
Cephalosporines	609 (37.40%)	459 (42.91%)	659 (30.83%)	1727 (36.10%)
Fluoroquinolones	277 (36.84%)	152 (35.50%)	190 (44.92%)	619 (39.70%)
Combination therapies	665 (33.74%)	398 (40.95%)	848 (34.90%)	1911 (35.87%)
Reserve	744 (29.25%)	424 (38.89%)	563 (31.99%)	1731 (32.57%)
Macrolides	321 (17.78%)	224 (19.98%)	298 (15.78%)	843 (17.51%)
Azoles	236 (17.40%)	134 (3.48%)	160 (16.84%)	530 (13.94%)
Carbapenems	198 (15.35%)	155 (13.56%)	179 (10.06%)	532 (12.86%)
Lincosamides	113 (13.07%)	9 (11.95%)	20 (8.47%)	142 (11.01%)
Urinary	235 (13.02%)	117 (10.44%)	479 (25.37%)	831 (17.26%)
Cyclines	528 (10.97%)	436 (13.83%)	604 (9.48%)	1568 (11.05%)
Other antibitoics	137 (7.59%)	85 (7.58%)	493 (26.11%)	715 (14.85%)
Glycopeptides	19 (6.26%)	3 (0.80%)	133 (1.06%)	155 (2.95%)
Other antifungals	314 (5.37%)	39 (0.62%)	318 (7.31%)	671 (5.03%)
Polyenes	97 (1.05%)	7 (0.27%)	138 (7.04%)	242 (3.22%)

Table S2.18. Incidence of microorganisms with multiple resistance patterns.

		2019	2021	2023	Total
Specific phenotypes	MRSA	55 (15.28%)	92 (36.08%)	38 (10.98%)	185 (19.25%)
	MRCoNS	62 (52.10%)	57 (81.43%)	63 (33.16%)	182 (48.02%)
	VRE	2 (1.96%)	4 (4.88%)	7 (3.48%)	13 (3.38%)
ESBL	Overall ESBL	554 (22.31%)	374 (22.41%)	395 (14.45%)	1323 (19.22%)
	Escherichia coli	183 (44.10%)	110 (35.14%)	143 (32.35%)	436 (37.26%)
	Klebsiella pneumoniae	152 (66.38%)	81 (59.12%)	147 (64.47%)	380 (63.97%)
	Pseudomonas aeruginosa	65 (29.02%)	68 (36.36%)	46 (23.35%)	179 (29.44%)
	Serratia marcescens	52 (91.23%)	35 (87.50%)	6 (27.27%)	93 (78.15%)
	Enterobacter spp.	39 (90.70%)	45 (97.83%)	16 (40.00%)	100 (77.52%)
	Proteus mirabilis	15 (17.65%)	2 (7.69%)	16 (24.62%)	33 (18.75%)
	Klebsiella spp.	11 (39.29%)	5 (41.67%)	5 (27.78%)	21 (36.21%)
	Morganella spp.	9 (100.00%)	2 (66.67%)	6 (85.71%)	17 (89.47%)
	Chryseobacterium spp.	7 (77.78%)	13 (92.86%)	0 (0.00%)	20 (86.96%)
	Citrobacter spp.	7 (50.00%)	5 (71.43%)	5 (38.46%)	17 (50.00%)
	Acinetobacter baumannii	6 (17.65%)	1 (4.55%)	1 (2.56%)	8 (8.42%)
	Other	4 (20.00%)	1 (5.88%)	0 (0.00%)	5 (10.87%)
	Sphingomonas paucimobilis	2 (11.11%)	1 (50.00%)	0 (0.00%)	3 (13.64%)
	Pseudomonas spp.	1 (20.00%)	2 (40.00%)	3 (20.00%)	6 (24.00%)
	Serratia spp.	1 (50.00%)	1 (16.67%)	0 (0.00%)	2 (22.22%)
	Acinetobacter spp.	0 (0.00%)	2 (25.00%)	1 (6.67%)	3 (9.09%)
CRO	Overall CRO	112 (4.51%)	147 (8.81%)	91 (3.33%)	350 (5.08%)
	Pseudomonas aeruginosa	58 (25.89%)	84 (44.92%)	30 (15.23%)	172 (28.29%)
	Klebsiella pneumoniae	14 (6.11%)	20 (14.60%)	45 (19.74%)	79 (13.30%)
	Proteus mirabilis	8 (9.41%)	1 (3.85%)	3 (4.62%)	12 (6.82%)
	Chryseobacterium spp.	7 (77.78%)	14 (100.00%)	0 (0.00%)	21 (91.30%)
	Acinetobacter baumannii	5 (14.71%)	1 (4.55%)	1 (2.56%)	7 (7.37%)
	Escherichia coli	5 (1.20%)	2 (0.64%)	3 (0.68%)	10 (0.85%)
	Sphingomonas paucimobilis	5 (27.78%)	1 (50.00%)	0 (0.00%)	6 (27.27%)
	Enterobacter spp.	4 (9.30%)	15 (32.61%)	1 (2.50%)	20 (15.50%)
	Klebsiella spp.	2 (7.14%)	1 (8.33%)	1 (5.56%)	4 (6.90%)
	Other	2 (10.00%)	1 (5.88%)	1 (11.11%)	4 (8.70%)
	Morganella spp.	1 (11.11%)	0 (0.00%)	0 (0.00%)	1 (5.26%)
	Pseudomonas spp.	1 (20.00%)	0 (0.00%)	1 (6.67%)	2 (8.00%)
	Acinetobacter spp.	0 (0.00%)	1 (12.50%)	3 (20.00%)	4 (12.12%)
	Proteus spp.	0 (0.00%)	1 (33.33%)	0 (0.00%)	1 (10.00%)
	Serratia marcescens	0 (0.00%)	4 (10.00%)	2 (9.09%)	6 (5.04%)
	Serratia spp.	0 (0.00%)	1 (16.67%)	0 (0.00%)	1 (11.11%)
MDR	Overall MDR	1750 (70.48%)	1076 (64.47%)	1864 (68.20%)	4690 (68.12%)
	Escherichia coli	321 (77.35%)	244 (77.96%)	369 (83.48%)	934 (79.83%)
	Klebsiella pneumoniae	226 (98.69%)	135 (98.54%)	224 (98.25%)	585 (98.48%)
	Staphylococcus aureus	207 (57.50%)	133 (52.16%)	308 (89.02%)	648 (67.43%)
	Pseudomonas aeruginosa	194 (86.61%)	128 (68.45%)	154 (78.17%)	476 (78.29%)
	Streptococcus pneumoniae	154 (77.39%)	58 (80.56%)	116 (62.37%)	328 (71.77%)
	CoNS	119 (100.00%)	63 (90.00%)	184 (96.84%)	366 (96.57%)

	Proteus mirabilis	82 (96.47%)	25 (96.15%)	60 (92.31%)	167 (94.89%)
	Streptococcus gr.A	66 (34.55%)	3 (21.43%)	76 (19.95%)	145 (24.74%)
	Enterococcus faecalis	57 (98.28%)	26 (100.00%)	53 (100.00%)	136 (99.27%)
	Serratia marcescens	56 (98.25%)	39 (97.50%)	20 (90.91%)	115 (96.64%)
	Enterobacter spp.	39 (90.70%)	45 (97.83%)	22 (55.00%)	106 (82.17%)
	Acinetobacter baumannii	31 (91.18%)	16 (72.73%)	16 (41.03%)	63 (66.32%)
	Stenotrophomonas maltophilia	31 (70.45%)	39 (46.99%)	28 (46.67%)	98 (52.41%)
	Enterococcus faecium	29 (100.00%)	39 (100.00%)	62 (95.38%)	130 (97.74%)
	Klebsiella spp.	28 (100.00%)	11 (91.67%)	18 (100.00%)	57 (98.28%)
	Other	18 (90.00%)	10 (58.82%)	5 (55.56%)	33 (71.74%)
	Sphingomonas paucimobilis	14 (77.78%)	1 (50.00%)	(0.00%)	15 (68.18%)
	Enterococcus spp.	13 (86.67%)	16 (94.12%)	72 (86.75%)	101 (87.83%)
	Chryseobacterium spp.	9 (100.00%)	14 (100.00%)	0 (0.00%)	23 (100.00%)
	Morganella spp.	9 (100.00%)	2 (66.67%)	7 (100.00%)	18 (94.74%)
	Acinetobacter spp.	8 (80.00%)	3 (37.50%)	11 (73.33%)	22 (66.67%)
	Citrobacter spp.	8 (57.14%)	5 (71.43%)	10 (76.92%)	23 (67.65%)
	Streptococcus spp.	7 (58.33%)	2 (66.67%)	11 (64.71%)	20 (62.50%)
	Candida parapsilosis	6 (9.23%)	(0.00%)	(0.00%)	6 (4.11%)
	Salmonella spp.	4 (100.00%)	4 (100.00%)	5 (33.33%)	13 (56.52%)
	Streptococcus gr.B	4 (80.00%)	3 (60.00%)	11 (100.00%)	18 (85.71%)
	Pseudomonas spp.	3 (60.00%)	4 (80.00%)	10 (66.67%)	17 (68.00%)
	Candida albicans	2 (1.64%)	1 (0.78%)	1 (0.74%)	4 (1.04%)
	Serratia spp.	2 (100.00%)	4 (66.67%)	(0.00%)	6 (66.67%)
	Candida spp.	1 (2.78%)	(0.00%)	2 (7.41%)	3 (3.00%)
	Candida tropicalis	1 (5.88%)	(0.00%)	(0.00%)	1 (2.56%)
	Proteus spp.	1 (20.00%)	3 (100.00%)	2 (100.00%)	6 (60.00%)
	Haemophilus influenzae	0 (0.00%)	0 (0.00%)	7 (53.85%)	7 (53.85%)
XDR	Overall XDR	245 (9.87%)	112 (6.71%)	188 (6.88%)	545 (7.92%)
	Klebsiella pneumoniae	84 (36.68%)	31 (22.63%)	90 (39.47%)	205 (34.51%)
	Pseudomonas aeruginosa	39 (17.41%)	30 (16.04%)	33 (16.75%)	102 (16.78%)
	CoNS	32 (26.89%)	17 (24.29%)	34 (17.89%)	83 (21.90%)
	Escherichia coli	28 (6.75%)	12 (3.83%)	17 (3.85%)	57 (4.87%)
	Serratia marcescens	23 (40.35%)	4 (10.00%)	4 (18.18%)	31 (26.05%)
	Acinetobacter baumannii	6 (17.65%)	(0.00%)	(0.00%)	6 (6.32%)
	Enterobacter spp.	6 (13.95%)	9 (19.57%)	7 (17.50%)	22 (17.05%)
	Morganella spp.	5 (55.56%)	(0.00%)	(0.00%)	5 (26.32%)
	Other	5 (25.00%)	(0.00%)	(0.00%)	5 (10.87%)
	Proteus mirabilis	5 (5.88%)	1 (3.85%)	1 (1.54%)	7 (3.98%)
	Chryseobacterium spp.	4 (44.44%)	4 (28.57%)	0 (0.00%)	8 (34.78%)
	Klebsiella spp.	3 (10.71%)	3 (25.00%)	1 (5.56%)	7 (12.07%)
	Pseudomonas spp.	3 (60.00%)	(0.00%)	(0.00%)	3 (12.00%)
	Acinetobacter spp.	1 (10.00%)	(0.00%)	(0.00%)	1 (3.03%)
	Staphylococcus aureus	1 (0.28%)	1 (0.39%)	1 (0.29%)	3 (0.31%)
