# Supplementary material for: The Impact of the COVID-19 Pandemic on Pediatric Microbial Resistance Patterns and Abandonment Rates in Western Romania—An Interdisciplinary Study
Source: Antibiotics (Basel). 2025 Apr 16;14(4):411. doi: 10.3390/antibiotics14040411 (PMC12024448; doi:10.3390/antibiotics14040411)
Supplement: Supplementary file 1 [file antibiotics-14-00411-s001.zip › supplement S3- abandonment details.pdf]

Table S3.1. Demographic data of abandoned patients

		2019	2023	p*
Sex	F	2 (50.00%)	2 (66.67%)	0.6831
	M	2 (50.00%)	1 (33.33%)	
Location	R	1 (25.00%)	2 (66.67%)	0.3074
	U	3 (75.00%)	1 (33.33%)	
Age group	Infant	4 (100.00%)	3 (100.00%)	NA
	Age median (IQR) **	0 (0-0)	0 (0-0.25)	0.1573
Ward	ICU	1 (20.00%)	1 (25.00%)	0.5386
	Nutritional recovery	3 (60.00%)	1 (25.00%)	
	Preterm	1 (20.00%)	2 (50.00%)	
Diagnosis	Prematurity	1 (14.29%)	2 (50.00%)	0.3780
	Malnutrition	5 (71.43%)	2 (50.00%)	
	Spina bifida	1 (14.29%)	0 (0.00%)	
	LoS median (IQR) **	16 (1-21)	25 (9-25)	0.6188
Samples	Blood	0 (0.00%)	1 (20.00%)	0.0841
	Nasal secretion	2 (20.00%)	0 (0.00%)	
	Otic secretion	1 (10.00%)	0 (0.00%)	
	Peritoneal fluid	0 (0.00%)	2 (40.00%)	
	Umbilical secretion	0 (0.00%)	1 (20.00%)	
	Urine	6 (60.00%)	1 (20.00%)	

*: Chi[2] result, unless stated otherwise, **: Mann-Whitney test, NA: not applicable

Table S3.2. Pathogen analysis for abandoned patients

	2019			2023		
	Abandonment	No Abandonment	IRR (95% CI; p)	Abandonment	No Abandonment	IRR (95% CI; p)
Enterococcus faecalis	10.00%	2.30%	4.34 (0.11-25.17; 0.2318)	0.00%	1.94%	NA
Enterococcus spp.	0.00%	0.61%	NA	20.00%	3.01%	6.65 (0.17-38.12; 0.1513)
Escherichia coli	10.00%	16.74%	0.6 (0.02-3.35; 0.6891)	0.00%	16.20%	NA
Klebsiella pneumoniae	30.00%	9.14%	3.28 (0.67-9.72; 0.0806)	40.00%	8.28%	4.01 (0.58-17.64; 0.0748)
Proteus mirabilis	10.00%	3.40%	2.94 (0.07-16.86; 0.3368)	0.00%	2.38%	NA
Pseudomonas aeruginosa	20.00%	8.98%	2.23 (0.27-8.14; 0.2911)	0.00%	7.22%	NA
Stenotrophomonas maltophilia	0.00%	1.78%	NA	40.00%	2.13%	18.81 (2.23-71.09; 0.0057 *)
Streptococcus pneumoniae	20.00%	7.97%	2.51 (0.30-9.19; 0.2389)	0.00%	6.82%	NA

*: statistically significant, NA: not applicable

Table S3.3. Logistic regression analysis in regards to demographics and hospitalization

Variable	Coefficient	Std. Error	Wald	p	Odds ratio	95% CI
Age	-1.53	1.07	2.07	0.1507	0.22	0.02-1.74
Age group= Adolescent	0.03	16693.38	-	1.0000	1.03	-
Age group= Preschool	-18.35	12958.43	-	0.9989	0.00	-
Age group= School	-11.33	13392.74	-	0.9993	0.00	-
Sex= F	0.01	0.58	-	0.9949	1.00	0.32-3.11
Location= R	-0.53	0.56	0.89	0.3444	0.59	0.19-1.75
LoS	0.02	0.01	3.96	0.0467 *	1.02	1.00-1.04
ICU/ NICU	0.39	0.72	0.30	0.5864	1.48	0.36-6.00
Preterm	2.71	0.88	9.43	0.0021 *	14.97	2.66-4.16
Malnutrition	3.83	0.67	33.12	<0.0001 *	46.02	12.4-9.53
GNB	1.04	0.63	2.75	0.0975	0.35	0.10-1.20
Constant	-6.78	0.83	67.41	<0.0001 *		
Overall fit p	<0.0001 *					
R^2	0.0123					

*: statistically significant, GNB: Gram-negative bacillus
